# Antioxidant Effects of Eugenol on Oxidative Stress Induced by Hydrogen Peroxide in Islets of Langerhans Isolated from Male Mouse

**DOI:** 10.1155/2020/5890378

**Published:** 2020-12-30

**Authors:** Ali Akbar Oroojan, Narges Chenani, Marzieh An'aam

**Affiliations:** ^1^Department of Physiology, Faculty of Medicine, Student Research Committee, Dezful University of Medical Sciences, Dezful, Iran; ^2^Student Research Committee, Faculty School of Paramedical Sciences, Dezful University of Medical Sciences, Dezful, Iran; ^3^Faculty of Medicine, Dezful University of Medical Sciences, Dezful, Iran

## Abstract

**Background:**

The antioxidant system in islets of Langerhans is weak, which can lead to diabetes. Meanwhile, the main component of cloves that produce antioxidant effects is eugenol. Accordingly, the present study was conducted to investigate the antioxidant effect of eugenol on oxidative stress induced by hydrogen peroxide (H_2_O_2_) in islets of Langerhans isolated from the male mice.

**Materials and Methods:**

In this experimental study, adult Naval Medical Research Institute (NMRI) mice (20-25 g) were prepared. The collagenase digestion method was used for dissecting the islets of Langerhans. H_2_O_2_ 50 *μ*M was administered for 30 min to induce oxidative stress, with 50, 100, and 200 *μ*M of eugenol employed for 2 hours before the administration of H_2_O_2_. The experimental groups were divided into five groups: (control, H_2_O_2_, and H_2_O_2_+eugenol 50, 100, and 200 *μ*M). Finally, the islet's lipid peroxidation and antioxidants levels were measured by the ELISA assay method.

**Results:**

Malondialdehyde (MDA), total antioxidant capacity (TAC), superoxide dismutase (SOD), and catalase (CAT) increased in all groups when compared to the control (*P* < 0.05). MDA diminished in H_2_O_2_+eugenol 50, 100, and 200 *μ*M (*P* < 0.01) groups versus the H_2_O_2_. TAC was elevated when eugenol 50, 100, and 200 *μ*M was administered in oxidative stress-induced islets (*P* < 0.001). Also, CAT increased in the H_2_O_2_+eugenol 50 (*P* < 0.05) group in comparison with the H_2_O_2_ group.

**Conclusions:**

In conclusion, H_2_O_2_ induced oxidative stress and lipid peroxidation in the islets, and administration of eugenol recovered these alterations by raising the level of TAC and CAT, while reducing MDA as a lipid peroxidation biomarker.

## 1. Introduction

Increased blood glucose triggers the onset of a series of cascading reactions, which eventually lead to increased production of free radicals and oxidative stress in various tissues such as the pancreas [1]. Numerous reports have shown that reactive oxygen species (ROS) can induce damage in cells and tissues [2]. Several endogenous antioxidant compounds including superoxide dismutase (SOD), glutathione, and catalase (CAT) protect cells against free radicals, especially ROS. Meanwhile, studies have shown a significant reduction in the amount of enzymatic and nonenzymatic antioxidants in the blood and cells during chronic diseases such as diabetes [3]. Oxidative stress refers to an imbalance between the production of oxygen free radicals and the body's antioxidant defense capacity [4]. Also, free radicals reduce the body's antioxidant activity causing enzyme activity disorder as well as increased lipid peroxidation [5]. When unsaturated fatty acids are exposed to free radicals, a series of chain reactions result in the formation of electron-friendly lipids and lipid peroxidation. Malondialdehyde (MDA) is one of the most toxic types of aldehydes, causing lipid and tissue damage due to lipid peroxidation [6]. The use of antioxidant compounds will play an important role in reducing the consequences of chronic diseases such as diabetes. In this regard, the administration of plant origin compounds is associated with fewer side effects [7]. It has been reported that medicinal plants and active ingredients extracted from them can play a protective role against oxidative stress and tissue damage via increasing the antioxidant activity of CAT and SOD enzymes. As the weakening of the antioxidant system in the pancreas or islets of Langerhans induces the complications of diabetes, it is possible to prevent the progression of this disease by strengthening the antioxidant defense system in diabetic cases [8–10]. Clove has several traditional therapeutic properties including medicinal antiseptic, analgesic, and antimicrobial effects. The main component of cloves that produce these effects is eugenol. Eugenol (4-allyl-2-methoxy phenol) is a phenolic compound with different applications in the preparation of dental materials, health products, beverages, and baked foods. This compound can function as an antioxidant and prevent free radical-mediated diseases such as cancer, inflammation, and type 2 diabetes mellitus (T2DM), as well as cardiovascular, neurodegenerative, and periodontal disease [11, 12]. It has also been revealed that polyphenols have neuroprotective effects in addition to the beneficial effects against cardiovascular disease, diabetes, and geriatric conditions. Administration of flavonoid compounds with vitamin E has shown a protective effect against Alzheimer's and Parkinson's diseases via reducing ROS overproduction [13]. Further, alpha-lipoic acid with antioxidant activities has a therapeutic effect against deltamethrin-induced hepatic and renal oxidative damages through inhibiting lipid peroxidation and scavenging free radicals [14]. Pancreatic islets are more susceptible to oxidative stress and free radicals than other cells due to their low antioxidant defense capacity. Also, antioxidants such as phenolic compounds or flavonoids make pancreatic islets stronger to conflict with oxidative stress, especially during diabetes [15, 16]. Thus, due to the antioxidant effects of eugenol and the susceptibility of the pancreatic islet to oxidative stress which can cause diabetes, the present study was conducted to examine the antioxidant effect of eugenol on oxidative stress induced by hydrogen peroxide (H_2_O_2_) in islets of Langerhans isolated from the male mouse.

## 2. Materials and Methods

### 2.1. Animal's Preparation

In this experimental study, adult Naval Medical Research Institute (NMRI) mice (20-25 g) were kept at a 12 : 12 hour light : dark cycle. The animals were treated in accordance with the principles and guidelines on animal care of Dezful University of Medical Sciences as reviewed by an ethics committee (IR.DUMS.REC.1398.022), as well as free access to tap water and commercial chow ad libitum.

### 2.2. Islet Isolation

The islets of Langerhans were isolated by the protocol for the isolation of islets from rodent pancreas using collagenase digestion. Briefly, the animals were anesthetized with ketamine/xylazine (70/10 mg/kg), and their pancreas removed and transferred to a Petri dish containing Krebs-bicarbonate buffer solution (Merck, Germany). Then, the separated pancreas was cut into 1 mm pieces and centrifuged at 100 ×g for 5 min. Krebs-bicarbonate buffer solution plus collagenase type P (1-2 mg/pancreas) (Roche, Germany) were added to the sediment of the centrifuged conical tube to separate the islets from exocrine tissue. Next, the conical tube was transferred to an incubator at 37°C with 800 oscillations for 5-10 min. Then, a cold Krebs-bicarbonate buffer was added to the conical tube to stop collagenase digestion and centrifuged at 500 ×g for 5 min. Finally, the isolated islets were transferred to a Petri dish and separated manually under a stereomicroscope [17].

### 2.3. Islets Induced Oxidative Stress and Treatment

To create oxidative stress in the isolated islets of Langerhans, H_2_O_2_ (50 *μ*M) was added to each islet's sample and incubated for 30 min. Then, the samples were centrifuged at 400 ×g for 10 min. In order to reduce the oxidative stress in islets, 50, 100, and 200 *μ*M of eugenol were added to each islet's sample and incubated for 2 hours at 37°C; after the incubation period, the oxidative-induced protocol with H_2_O_2_ (50 *μ*M) was repeated. Finally, the supernatant of each microtube was stored at -70°C until the experimental measurement. Each microtube contained 7 islets, and the number of samples is repeated 6 times for each group [18, 19].

### 2.4. Grouping

*Group 1*: control

*Group 2*: H_2_O_2_ (it received 50 *μ*M of H_2_O_2_ for 30 min)

*Group 3*: H_2_O_2_+eugenol (50 *μ*M) (it received 50 *μ*M of eugenol for 2 hours after which H_2_O_2_ (50 *μ*M) was added to each sample for 30 min)

*Group 4*: H_2_O_2_+eugenol (100 *μ*M) (it received 100 *μ*M of eugenol for 2 hours after which H_2_O_2_ (50 *μ*M) was added to each sample for 30 min)

*Group 5*: H_2_O_2_+eugenol (200 *μ*M) (it received 200 *μ*M of eugenol for 2 hours after which H_2_O_2_ (50 *μ*M) was added to each sample for 30 min)

### 2.5. Lipid Peroxidation and Antioxidant Measurements

To measure the level of MDA, total antioxidant capacity (TAC), SOD, and CAT in the isolated islets of Langerhans, the ELISA method as well as specific commercial kits (Teb Pazhouhan Razi, Iran) was used.

### 2.6. Statistical Analysis

The results were statistically analyzed using the Statistical Package for Social Sciences (SPSS) software with one-way analysis of variance (ANOVA), followed by post hoc least significant difference (LSD) tests. All results were represented as mean ± standard error (SE) where *P* was considered significant at less than 0.05 in all experiments.

## 3. Results

### 3.1. The Role of Eugenol in Islet's Lipid Peroxidation

**Figure 1:**
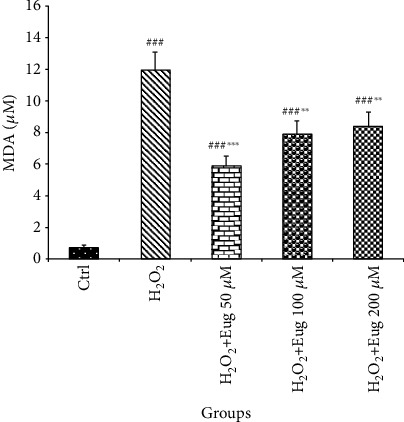
Effects of H_2_O_2_ and eugenol on the MDA level of islets of Langerhans. Data are expressed as the mean ± SE, *n* = 6 (7 islets in each sample). ^###^*P* < 0.001 significantly different with the control group; ^∗∗^*P* < 0.01 and ^∗∗∗^*P* < 0.001 significantly different with the H_2_O_2_ group. Eug: eugenol.

The results of this study showed that MDA increased in all groups when compared to the control (*P* < 0.001). This lipid peroxidation variable diminished in H_2_O_2_ plus eugenol 50 (*P* < 0.001), 100, and 200 *μ*M (*P* < 0.01) administrated groups versus the H_2_O_2_ group (Figure 1).

### 3.2. Antioxidant Activity of Eugenol in Isolated Islets of Langerhans

**Figure 2:**
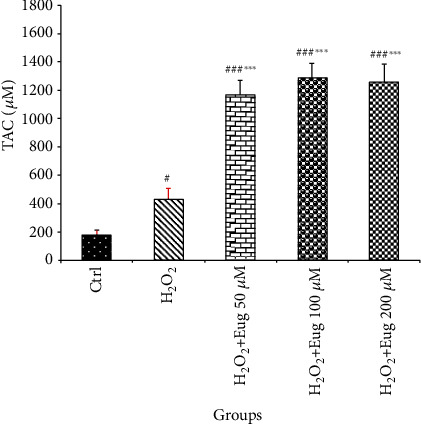
Effects of H_2_O_2_ and eugenol on the TAC level of islets of Langerhans. Data are expressed as the mean ± SE, *n* = 6 (7 islets in each sample). ^#^*P* < 0.05 and ^###^*P* < 0.001 significantly different with the control group; ^∗∗∗^*P* < 0.001 significantly different with the H_2_O_2_ group. Eug: eugenol.

The islets' level of TAC increased in H_2_O_2_ (*P* < 0.05), as well as H_2_O_2_ plus eugenol 50, 100, and 200 *μ*M (*P* < 0.001) groups compared with the control. This variable revealed a significant increase following eugenol 50, 100, and 200 *μ*M (*P* < 0.001) administration under oxidative stress islets compared to the H_2_O_2_ group (Figure 2).

**Figure 3:**
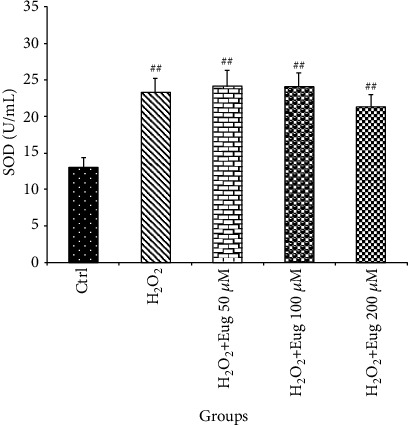
Effects of H_2_O_2_ and eugenol on the SOD level of islets of Langerhans. Data are expressed as the mean ± SE, *n* = 6 (7 islets in each sample). ^##^*P* < 0.01 significantly different with the control group. Eug: eugenol.

The results indicated that the SOD level rose in all groups when compared to control (*P* < 0.01). However, this antioxidant enzyme did not show any significant alteration across the H_2_O_2_-administered groups (Figure 3).

**Figure 4:**
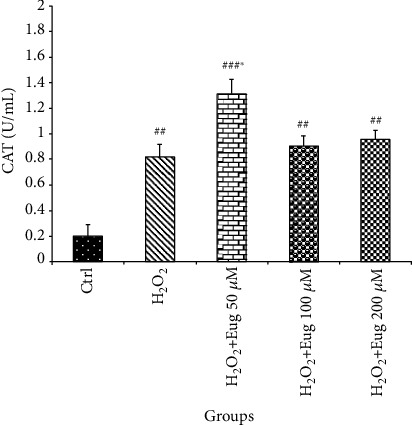
Effects of H_2_O_2_ and eugenol on the CAT level of islets of Langerhans. Data are expressed as the mean ± SE, *n* = 6 (7 islets in each sample). ^##^*P* < 0.01 and ^###^*P* < 0.001 significantly different with the control group; ^∗^*P* < 0.05 significantly different with the H_2_O_2_ group. Eug: eugenol.

CAT level increases in H_2_O_2_ (*P* < 0.01) and H_2_O_2_ plus eugenol 50 (*P* < 0.001), 100, and 200 *μ*M (*P* < 0.01) groups compared to the control. Also, this enzyme was elevated in the H_2_O_2_ plus eugenol 50 *μ*M (*P* < 0.05) group in comparison with the H_2_O_2_ group (Figure 4).

## 4. Discussion

The results of the present study showed that the induction of oxidative stress by H_2_O_2_ would lead to higher MDA levels in the isolated islets of Langerhans. However, H_2_O_2_ could increase TAC, SOD, and CAT, but this increasing effect could not improve the damage induced by oxidative stress and lipid peroxidation in the islets. The administration of eugenol decreased MDA along with increase TAC and CAT in pancreatic islets under oxidative stress condition induced by H_2_O_2_. Also, the effect of low doses of eugenol on improving the damage induced by oxidative stress was greater than that of other doses. This effect can be attributed to the fact that a low dose of eugenol resulted in a significant increase in catalase as well as in further reduction in MDA in the isolated islets.

Pancreatic *β* cells are sensitive to oxidative stress where ROS may play a central role in *β* cell death causing T1DM. Also, this disorder has a secondary pathogenic role in the development of T2DM. On the other hand, it was demonstrated that antioxidants such as phenolic compounds reduced this complication. H_2_O_2_ has multiple effects on *β* cells including metabolic inhibition and enhancing plasma membrane permeability, impairing glucose metabolism, and reducing insulin secretion [20]. Oxidative stress can increase the production of hydrogen peroxide and ketoaldehydes through spontaneous glucose oxidation in pancreatic *β* cell, causing type 1 and type 2 diabetes [21]. In addition, several phenolic compounds such as quercetin, catechin, and ascorbic acid could improve the antioxidant defense in various cells. Further, these flavonoids recovered H_2_O_2_-induced proliferation inhibition in *β* cell, and it was suggested that these antioxidants can be used in T2DM to increase *β* cell proliferation [22]. Diabetes mellitus disrupts the oxidant/antioxidant balance in healthy tissues such as the heart. This chronic disease increases the main cardiac variables including plasma troponin I and creatine kinase-MB (CK-MB) via reducing the antioxidant enzyme level causing cardiac damages. Also, it was demonstrated that thymoquinone as a potent antioxidant phytochemical ameliorates the cardiac injuries in diabetic rats via reducing oxidative stress and can also increase cell survival [23]. Overproduction of ROS is a common pathway of diverse pathogenic mechanisms of diabetic complications. Bioflavonoids as antioxidants are therapeutic options against diabetes mellitus and its related complications. Thus, maintaining glycemic control, blocking the pathways of free radical production, and increasing the antioxidants enzyme level can be a promising approach to the treatment of hyperglycemia-induced diabetes complications in humans [24]. Accordingly, present results suggested that eugenol may play a critical role in the treatment of DM through its antioxidant properties and reducing lipid peroxidation in pancreatic islets. Nevertheless, future clinical studies are required to demonstrate this recommendation.

It was revealed that antioxidant agents such as polyphenols or flavonoids scavenge free radicals depending on their concentration [11]. Further, these compounds can promote the generation of free radicals such as H_2_O_2_ at higher concentrations [20]. So, in accordance with a previous study, the reason for the greater effect of eugenol on low doses is the influence of this dose on reducing oxidative stress and improving the performance of antioxidant defenses in isolated pancreatic islets.

## 5. Conclusion

In conclusion, H_2_O_2_ induced oxidative stress and lipid peroxidation in the isolated islet of Langerhans, and administration of eugenol recovered these alterations by raising the level of TAC and CAT and reducing MDA as a lipid peroxidation biomarker. Also, among the administered concentrations of eugenol, a low dose of this phenolic compound was more potent to produce a therapeutic effect in the function of antioxidant enzymes and reducing the oxidative stress of the pancreatic islet. Hence, this dose of eugenol could be used to treat diabetes by improving the function of *β* cells in the islets of Langerhans, but a future clinical study is required to clarify this finding.

## Data Availability

The data are available from the corresponding author upon request.
